# TOT 8/4: A Way to Standardize the Surgical Procedure of a Transobturator Tape

**DOI:** 10.1155/2016/4941304

**Published:** 2016-02-15

**Authors:** Sebastian Ludwig, Martin Stumm, Peter Mallmann, Wolfram Jager

**Affiliations:** Department of Obstetrics and Gynecology, Division of Urogynecology and Pelvic Floor Surgery, University of Cologne, Kerpener Strasse 34, 50931 Cologne, Germany

## Abstract

Suburethral tapes are placed “tension-free” below the urethra. Several studies reported considerable differences of the distance between urethra and tape. These distances ranged from 1 to 10 mm amongst different patients. This either caused urethral obstruction or had no effect on urinary incontinence. Therefore, we decided to standardize the procedure by placing a Hegar dilator of 8-millimeter diameter in the urethra and another Hegar dilator of 4-millimeter diameter between the urethra and the tape during transobturator tape placement. Using that simple technique, which we named “TOT 8/4,” we observed that 83% of the tapes were placed in the desired distance between 3 and 5 millimeters below the urethra.

## 1. Introduction

During recent years, suburethral tapes have become the standard treatment for stress urinary incontinence (SUI) [1]. Different ways of positioning the tapes have been introduced either behind the symphysis (TVT) or through the foramen obturatum (TOT) [2, 3]. These tapes are supposed to be placed “tension-free” [2].

Using these techniques, more than 80% of the patients became continent [4]. Yet, some of our patients complained about a diminished urine flow after the operations. We hypothesized that this was caused by urethral obstruction due to a diminished distance between tape and urethra.

According to the original description, the ideal distance between urethra and tape was described as a “Metzenbaum scissor.” This describes a distance of 3 to 5 millimeters (mm) [5]. However, publications argue that up to 60% of tapes were placed in less than 3 mm distance to the urethra. Furthermore, that series reports obstructive complications amongst patients [6].

We therefore concluded that the diminished distance between urethra and tape was a problem of general scope and not only limited to our institution. In order to solve this problem, we decided to standardize the placement of the transobturator tape.

We now report on a simple surgical procedure to standardize the distance between transobturator tape and urethra.

## 2. Material and Methods

All patients with an indication for a transobturator tape (TOT) after urogynecological examinations were eligible for this study. Thereby, our institution made use of the TOT technique according to Delorme [3]. Two Hegar dilators, usually utilized for cervical dilatation, were used. The diameters of these were 8 millimeters (mm) (Hegar 8 dilator) and 4 mm (Hegar 4 dilator). Additionally, transobturator tapes made of polyvinylidene fluoride (PVDF) were used (Dynamesh-SIS soft, FEG Textiltechnik mbH, Aachen, Germany).

The authors undertook the operations. The experiences with TOT operations, expressed as total numbers of TOT operations prior to the study, differed between the surgeons: senior author >500 operations, first author: 50 operations, and second coauthor >200 operations.

Main outcome measure was the distance between urethra and “TOT 8/4” tape four months after surgery. Since 2013, we have been using the TOT 8/4 surgical technique to the present day.

## 3. TOT 8/4 Surgical Technique

Initially, the bladder was emptied and 50 mL of blue saline solution was refilled into the bladder. This was done, in order to detect injuries of the bladder. After palpating the location of the bladder neck, the Foley catheter was removed and the vaginal epithelium was incised one centimeter below the external urethral orifice of about 3 cm in length. The paraurethral tissue was distanced by sharp and blunt dissection until the obturator canal was palpated. Thereafter, the TOT tape was placed in the outside-in direction, in accordance with Delorme, always making use of the small trocar [3].

**Figure 1:**
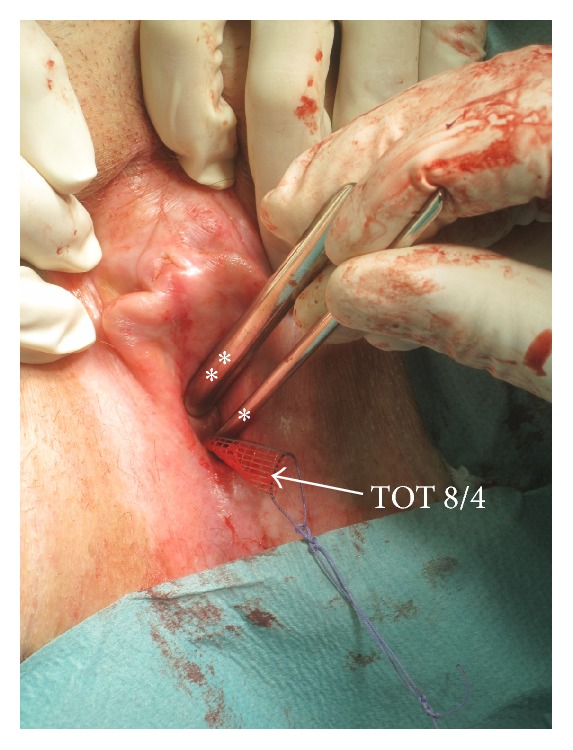
Standardization of the transobturator tape placement in the TOT 8/4 surgical technique. While placing the suburethral tape according to Delorme, one Hegar 8 dilator (*2 white asterisks*) was placed intraurethrally and one Hegar 4 dilator (*1 white asterisk*) was placed suburethrally between urethra and implanted tape. When tightening the tape, the distance between tape and urethra remained defined because the two Hegar dilators remained in the shown position.

Subsequently, a Hegar 8 dilator was placed into the urethra and one Hegar 4 dilator was placed suburethrally between the tape and the urethra. The surgeon held the Hegar dilators in parallel position and the surgical assistant tightened the TOT until the tape could not be “advanced” anymore (Figure 1).

**Figure 2:**
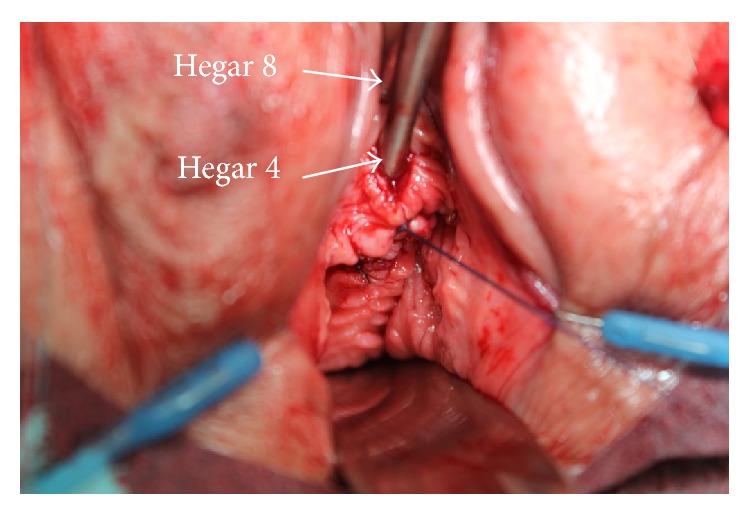
After TOT 8/4 placement a vaginal speculum was placed exposing the anterior vagina. The anterior vaginal epithelium was closed with an absorbable monofilament suture. While suturing, the Hegar 8 dilator was left intraurethrally and the Hegar 4 suburethrally in order to avoid tape displacement.

The external tape ends were cut close to the skin and covered with Steri-Strips. The vaginal epithelium was closed by using a vicryl 3-0 absorbable monofilament, running suture. Both Hegar dilators remained in parallel position during vaginal epithelium suturing (Figure 2).

## 4. Transvaginal Ultrasound Measurements 

All patients were asked to be present at the outpatient clinic 4 weeks and 4 months after surgery.

**Figure 3:**
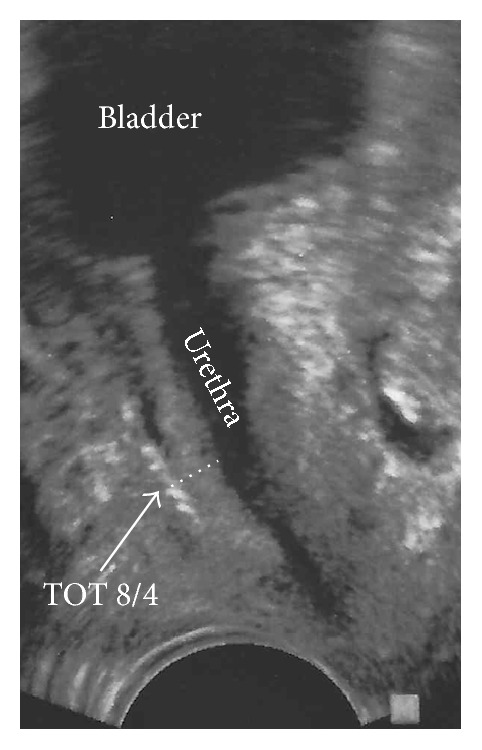
Transvaginal ultrasound in median sagittal view. A vaginal probe was used without exerting pressure to the midurethra. The center of the placed echogenic tape (TOT 8/4) is marked by the* white arrow head*. The shortest measured distance between the center of the tape and the lower part of the urethra is marked by the* broken line*.

Transvaginal ultrasound was performed under standardized conditions using a vaginal 4–9 MHz probe (SonoAce X8, Samsung Medison Co., Ltd.). Patients were in semisitting position and the bladder was filled with a volume of 300 mL. The vaginal probe was positioned in the area of the vaginal introitus at the level of the external urethral orifice at median sagittal position under the slightest pressure possible. The same examiner (first author) performed ultrasound examinations. Distances were measured in millimeters (mm). The shortest distance between the center of the tape and the lower part of the urethra was measured by drawing a right angle line on the border of the periurethral muscle reaching to the center point of the high echogenic tape (Figure 3).

## 5. Results 

Since 2013, 35 patients were operated on by making use of the TOT 8/4 surgical technique. In comparison, 46 women, who were so far operated on by the standard “tension-free” TOT, served as a “control.”

**Figure 4:**
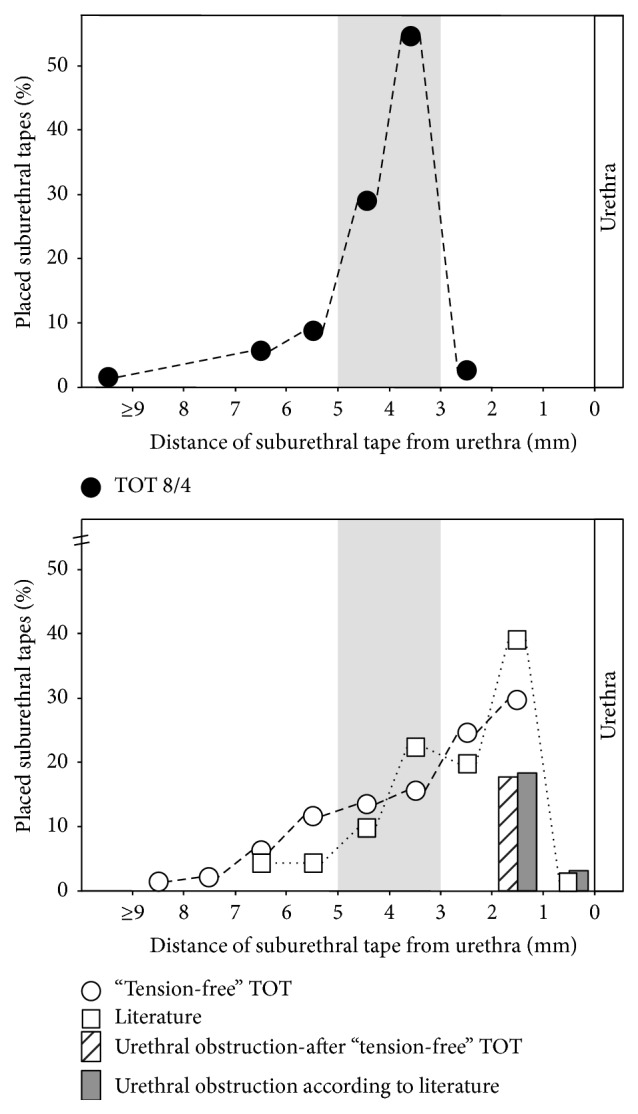
Distances in millimeters (mm) between the suburethral tapes and the urethra. With the TOT 8/4 technique (*black dots*), 83% of tapes were placed within a tape-urethra distance of 3–5 mm (*light grey marked area*). Only 28% of tapes of our so far standard “tension-free” TOT technique (*white dots*) were found in that area. According to the literature, up to 60% of suburethral tapes were placed in less than 3 mm distance to the urethra (*white squares*) [6]. Urethral obstruction was observed in about 20% of patients after “tension-free” TOT (*grey dotted columns*) and according to the literature (*dark grey columns*). No urethral obstruction was observed after TOT 8/4.

Using the TOT 8/4 surgical technique, 29 of the 35 tapes (83%) were placed within a distance of 3.1 to 4.9 mm, below the urethra (Figure 4). Out of the 46 patients who were operated on by the standard “tension-free” TOT in our institution, only 13 patients (28%) fell within that scope (Figure 4). In 23 cases out of the 46 patients (51%), the tapes were placed <3 mm and in 10 cases (21%) were placed in >5 mm distance to the urethra. Performing the TOT 8/4 surgical procedure, in the case of 1 patient, a distance of <3 mm to the urethra was measured, and in 5 patients a distance of >5 mm was measured.

Incontinence cure rates in TOT 8/4 patients were in exactly the same range as in the standard “tension-free” TOT (82% and 83%). During TOT 8/4 surgical procedure, no major hemorrhage events occurred and 4 months after surgery no urethral obstruction was observed.

## 6. Discussion

The original description of the TVT had a precise guidance on how to establish the desired distance between tape and urethra. Ulmsten et al. described the distance to achieve as “placing a Foley 16-Charrière catheter in the urethra and a Metzenbaum scissor between urethra and tape” [2]. Delorme defined the distance between TOT and urethra as a “visible distance of few millimeters” [3].

The “Metzenbaum scissor” blades measured a diameter of 3 to 5 mm.

We therefore decided that the distance between urethra and tape according to the description of Ulmsten should be in the range of 4 mm. Thus, a Hegar 4 dilator with a diameter of 4 mm was used in order to establish this distance during TOT surgery.

Since urethral obstruction should be prevented, we initially decided to choose a urethral catheter with a larger diameter. Ulmsten et al. chose a Foley 16-Charrière catheter with a diameter of 5.3 mm [2]. We therefore applied a Hegar 8 dilator, which has a diameter of 8 mm and which resembled a Foley 24-Charrière catheter [7].

By using these two Hegar dilators during the placement and the tightening of the transobturator tape, we could achieve a high degree of standardization (83% of tapes placed between 3 and 5 mm below the urethra). Hence, we named the technique “TOT 8/4.”

With the standard “tension-free” TOT, we previously placed about 51% of all transobturator tapes closer than 3 mm to the urethra. This caused signs of urethral obstruction (diminished urine flow) in several patients. We did not expect such a high number of patients with such a small distance between the tape and the urethra. However, we realized that also other authors reported the same outcome in regard to this issue [6, 8]. We therefore assumed that the ideal placement of the tape was a general problem of the technique itself.

After our first operations, using the TOT 8/4 procedure, we were expecting a distance between urethra and tape of at least 4 mm. However, in several cases, the distance was <4 mm. This was first realized amongst patients who exhibited a previous anterior colporrhaphy with vaginal skin resection. We hypothesized that the closure of the vaginal epithelium above the tapes could become too tight and was pushing the tape closer to the urethra. Therefore, we left the two Hegar dilators in place (intraurethral and suburethral) during the closure of the vaginal epithelium (Figure 2). They were removed before the final closing stitch. We soon realized that closing the anterior vaginal epithelium with the dilators in situ became more difficult with some of the patients who had a vaginal skin resection during a previous anterior colporrhaphy. The same difficulties were observed when the surgeon decided to take more vaginal skin into the suture. As a matter of fact, most of these patients featured a distance of 3 mm to 4 mm between urethra and tape, after the surgery.

The reasons for distances greater than 5 mm between tape and urethra remained unclear. We documented a hemorrhage of the paravaginal veins in 4 of 5 of these patients that led to a vaginal package for 2 hours after surgery. Since these intraoperative minor hemorrhage events never became a clinical problem afterwards, we did not further control the development of a hematoma after surgery. We hypothesize that the increased distance between tape and urethra was caused by the hematoma formation in these patients. Interestingly, we noted that all but one of these patients (distance 10 mm) were nevertheless continent after surgery. We assume that the new fibrotic tissue between tape and urethra exerts the same effect as a correctly placed tape.

These data were based on a follow-up 4 months after surgery. We cannot exclude the fact that the position of the tapes changes with increasing time after surgery. It is known that particularly the polyethylene tapes shrink with time. We therefore used polyvinylidene fluoride (PVDF) tapes which have a minor tendency for shrinkage [9].

The TOT 8/4 surgical technique, using a Hegar 8 and Hegar 4 dilator, resulted in a standardized distance between tape and urethra with the range of 3.1 to 4.9 mm in 83% of the cases. Thereby, the cure rate of SUI remained the same result as with the standard TOT; however, no urethral obstruction was observed in this study using the TOT 8/4.

With this practical tool even younger colleagues can easily learn the correct placement of the tapes. Prospectively, the identical technique allows the analysis and comparison of treatment studies between different centers.
